# Patterns of rangeland productivity and land ownership: Implications for conservation and management

**DOI:** 10.1002/eap.1862

**Published:** 2019-02-27

**Authors:** Nathaniel P. Robinson, Brady W. Allred, David E. Naugle, Matthew O. Jones

**Affiliations:** ^1^ W.A. Franke College of Forestry and Conservation University of Montana Missoula Montana 59812 USA; ^2^ Numerical Terradynamic Simulation Group University of Montana Missoula Montana 59812 USA

**Keywords:** ecosystem services, land tenure, net primary production, private land, public land, rangelands

## Abstract

Rangelands cover 40–50% of the Earth's terrestrial surface. While often characterized by limited, yet variable resource availability, rangelands are vital for humans, providing numerous ecosystem goods and services. In the conterminous United States (CONUS), the dominant component of rangeland conservation is a network of public rangelands, concentrated in the west. Public rangelands are interspersed with private and tribal rangelands resulting in a complex mosaic of land tenure and management priorities. We quantify ownership patterns of rangeland production at multiple scales across CONUS and find that both total production and average productivity of private rangelands is more than twice that of public and tribal rangelands. At finer scales, private rangelands are consistently more productive than their public counterparts. We also demonstrate an inverse relationship between public rangeland acreage and productivity. While conserving acreage is crucial to rangeland conservation, just as critical are broad‐scale ecological patterns and processes that sustain ecosystem services. Across CONUS, ownership regimes capture distinct elements of these patterns and services, demonstrated through disparate production dynamics. As ownership determines the range of feasible conservation actions, and the technical and financial resources available to implement them, understanding ownership‐production dynamics is critical for effective and sustained conservation of rangeland ecosystem services.

## Introduction

*… conservation will ultimately boil down to rewarding the private landowner who conserves the public interest*. —Leopold 1934

Rangelands are often described as uncultivated land dominated by grasses, forbs, and shrubs and suitable for grazing animals, inclusive of both livestock and wildlife (Briske 2017). Extensive and important systems, rangelands cover 40–50% of the Earth's ice‐free land surface and provide vital ecosystem services (Sala et al. 2017): rangelands store approximately 10–30% of the world's terrestrial carbon (Derner et al. 2006, Booker et al. 2013), are a source of water and animal protein for roughly two billion people (Havstad et al. 2009), and are rich in biodiversity (Havstad et al. 2009). While rangelands occur globally across wide ranges of climatic conditions, many intact rangelands (i.e., those not cultivated or otherwise developed) occur in regions with limited water and nutrient availability. Moreover, rangelands are coupled socioecological systems, shaped through interdependent land use practices and ecological processes (Havstad et al. 2009, Huntsinger and Sayre 2017). As human populations and their subsequent demand for resources increase, rangelands are undergoing rapid alterations (Allred et al. 2015). The conservation of rangeland systems for both their ecological and societal values is largely dependent on maintaining the overall patterns and processes that occur across multiple spatiotemporal scales (Fuhlendorf et al. 2012, Sayre et al. 2012).

**Figure 1:**
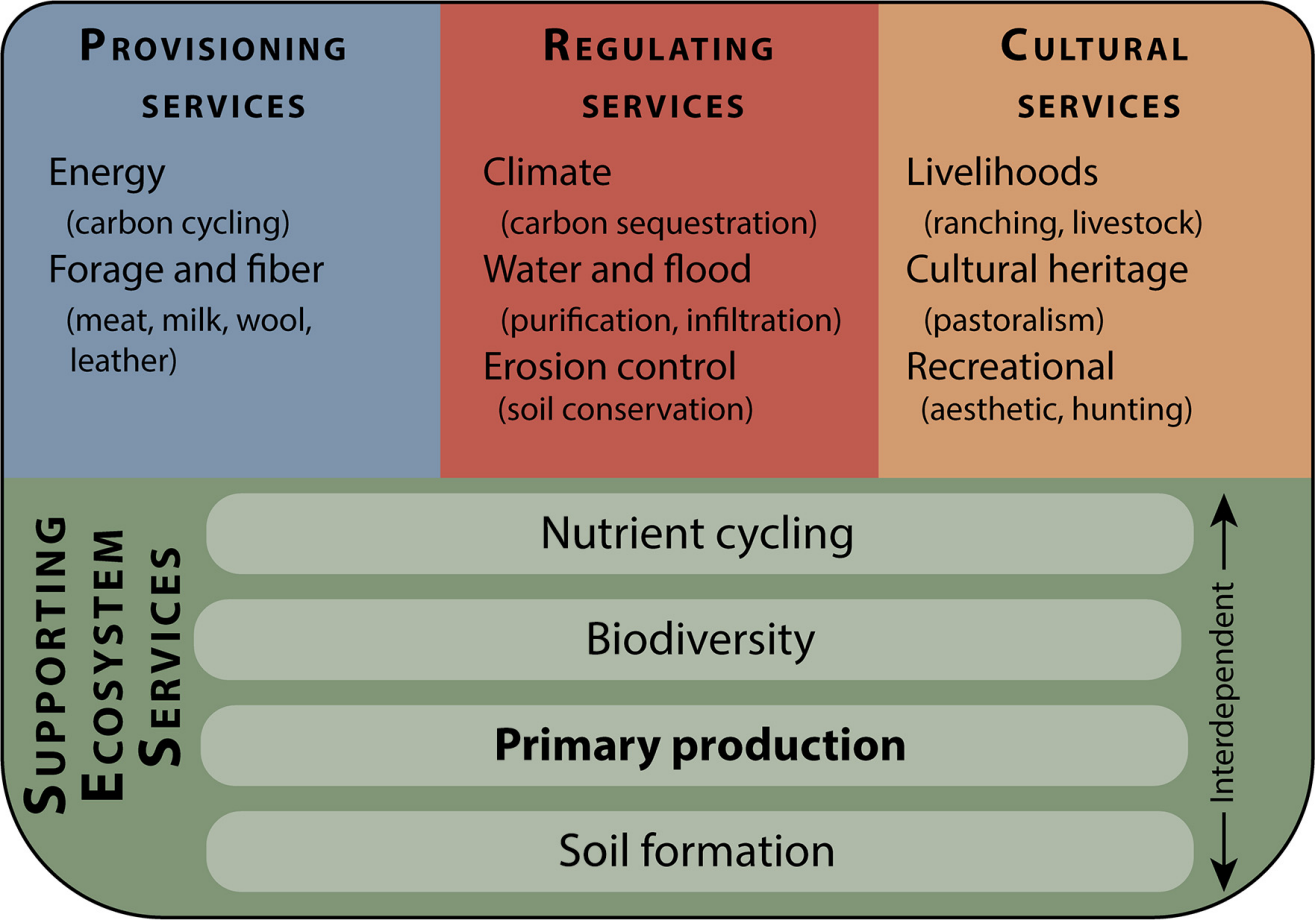
Ecosystem service categories as specified by the Millennium Ecosystem Assessment (Alcamo et al. 2003). Examples provided are relevant to rangelands and primary productivity, showing how primary productivity, as a supporting ecosystem service, is foundational for the provisional, regulating, and cultural services on rangelands. Figure adapted from Sala et al. (2017).

A fundamental driver of ecological and social dynamics across rangeland systems globally is net primary productivity (NPP). NPP quantifies the assimilation of CO_2_ into biomass through photosynthesis (Roy et al. 2001). As the entry point of carbon into ecosystems and the ultimate source of energy for all terrestrial species, it is considered a supporting ecosystem service, a category that includes ecosystem services that are necessary for the production of all other ecosystem services (Fig. 1; Alcamo et al. 2003). As a supporting ecosystem service, NPP directly supports the most familiar ecosystem services of rangelands: forage for meat, milk, wool, and leather (Sala et al. 2017). Even with low NPP compared to other ecosystems, rangelands support more herbivore biomass than any other terrestrial biome, making them important systems for livestock production and wildlife populations (Frank et al. 1998). Understanding productivity dynamics at broad and fine scales is essential in maintaining functioning landscapes that provide this and other numerous ecosystem services. Rangeland NPP is influenced by processes occurring at multiple scales, from broad climate patterns, to landscape‐level disturbance regimes, and to localized fine‐scale land‐use practices, and management actions. While broad‐scale patterns of rangeland NPP exist, regional and local patterns can be highly variable across space and time (Piao et al. 2009). The animals (wild or domestic) that rangelands support possess the mobility to exploit the spatiotemporal variability of vegetation productivity and resource availability across landscapes and through time (Middleton et al. 2018).

As vast acreage of U.S. rangelands is in the public domain (~39%) and have been a dominant focus and priority of rangeland conservation (Charnley et al. 2014), it is tempting to assume that the socioecological integrity of rangelands is adequately conserved across the conterminous United States (CONUS). Ownership patterns, however, reflect the historic socioeconomic and political conditions that encouraged private parties, both corporate and individual, to settle and develop the expanding U.S. territories over the 19th and early 20th centuries (Sheridan and Sayre 2014). Lands suitable for agriculture and industry—lands with available water, good soils, and accessible terrain—were rapidly privatized (Scott et al. 2001, Knight 2007, Talbert et al. 2007). In the western United States, large areas with scarce water, poor soils, and rugged terrain were left unsettled and eventually became part of the public domain. Because of this, it has long been surmised, though not quantified, that private rangelands exhibit disproportionately higher vegetation productivity than their public counterparts (Huntsinger and Hopkinson 1996, Knight 2007). If, in fact, true and private rangelands bare the bulk of productivity, private rangeland conservation efforts and paradigms will need equal attention and support as their public counterparts in order to better capture and conserve the ecological integrity of U.S. rangeland systems.

As land‐use practices and management regimes (e.g., grazing regime, conversion, development, restoration) can influence rangeland productivity dynamics, the people or institutions with the capacity to drive land‐use and management decisions have a pivotal role in rangeland conservation and management. In the United States, land ownership largely determines the feasibility of conservation and management actions, and the resources (e.g., technical and financial) available for their implementation. The public land system across federal, state, and local jurisdictions is a central component of U.S. rangeland conservation (Scott et al. 2001), managed through a rich history of policy and regulation. In contrast, top‐down policy and regulation (e.g., endangered species, hazardous or toxic substances) are poorly received by private and tribal land managers as a primary mechanism for conservation. Instead, voluntary and incentive‐based strategies are emerging as innovative mechanisms to accomplish conservation of U.S. private rangelands, commonly implemented through community partnerships (Duvall et al. 2017); tax incentives (e.g., conservation easements; Copeland et al. 2013); and technical and financial support from non‐governmental organizations and federal agencies (e.g., Natural Resources Conservation Service; Copeland et al. 2014). Private lands are the interstitial tissue that binds together into one integrated system the vast network of public rangelands. For a cohesive and broad‐scale rangeland conservation strategy to materialize, more must be known about the inherent variability in productivity among divergent ownerships.

Quantifying the ownership of rangeland productivity will provide greater understanding of (1) who (private or public) has stewardship of this ecosystem service and decides the management that can occur therewith; and (2) the technical and financial resources available and targeted for such management. To this end, we examine the ownership patterns of rangeland productivity across CONUS at multiple scales. Our objectives are (1) to quantify total production and average productivity across private, public, and tribal rangelands of the conterminous United States and (2) to examine the temporal trends of private, public, and tribal rangeland productivity.

## Methods

To examine production‐ownership relationships, we used a new high‐resolution NPP data set specifically developed for CONUS (Robinson et al. 2018). This data set, based on the MOD17 NPP model (Running and Zhao [Link]), uses optical remote sensing, meteorological data, and plant functional types to estimate the total amount of carbon allocated to plant tissue (above‐ and belowground) after accounting for losses due to autotrophic respiration. The NPP data set we use incorporates high‐resolution (30 m) Landsat estimates of vegetation dynamics along with high‐resolution land cover and meteorological data sets specific to CONUS and is well suited for monitoring the spatiotemporal variability of NPP across ownership, land‐use, and management regimes at ecologically relevant scales. We obtained land ownership from the Protected Areas Database of the US (PAD‐US CBI edition, version 2), a GIS data set containing polygons of land ownership across CONUS, designated as federal, state, local, tribal, and private (Conservation Biology Institute 2012). We classified ownership into three broad categories: public (aggregating federal, state, and local ownership), tribal (Native American reservations) and private. The PAD‐US represents ownership as of 2016; we assumed the transfer of land across the three major categories to be minimal during our study period (1993–2016). We used the 30‐m National Land Cover Database (NLCD; Homer et al. 2007, 2015, Fry et al. 2011) to disaggregate production–ownership results across rangelands. For this analysis, rangelands were delineated solely by land cover, as the combined shrubland and grassland classes from the NLCD. Croplands, pasture/hay, and built‐up (e.g., urban) areas were excluded from the analysis. For a given year, land cover from the closest subsequent NLCD year (2001, 2006, or 2011) was used.

To assess the overall ownership patterns of terrestrial production across CONUS rangelands, we calculated total production and average productivity annually from 1993 to 2017 for each ownership category at the CONUS scale. Total production is the cumulative amount of carbon allocated to plant tissue annually over a given area, often measured in Tg (10^12^) of carbon, while average productivity is the mean rate of allocation over a given area (kg C·m^−2^·yr^−1^). To assess temporal trends in rangeland productivity, we used simple linear regression between productivity and time. Additionally, we quantified the ownership–production relationships at the EPA Level II ecoregion and state scale. Dynamics at the state scale are important, as states represent relevant jurisdictional and management boundaries for both public and private land. We used Spearman's rank‐order correlation analysis to test for a correlation between the acreage of public lands and the average productivity of the public lands. All analyses were done in Google Earth Engine (Gorelick et al. 2016) and R (R Core Team 2015).

## Results and Discussion

**Figure 2:**
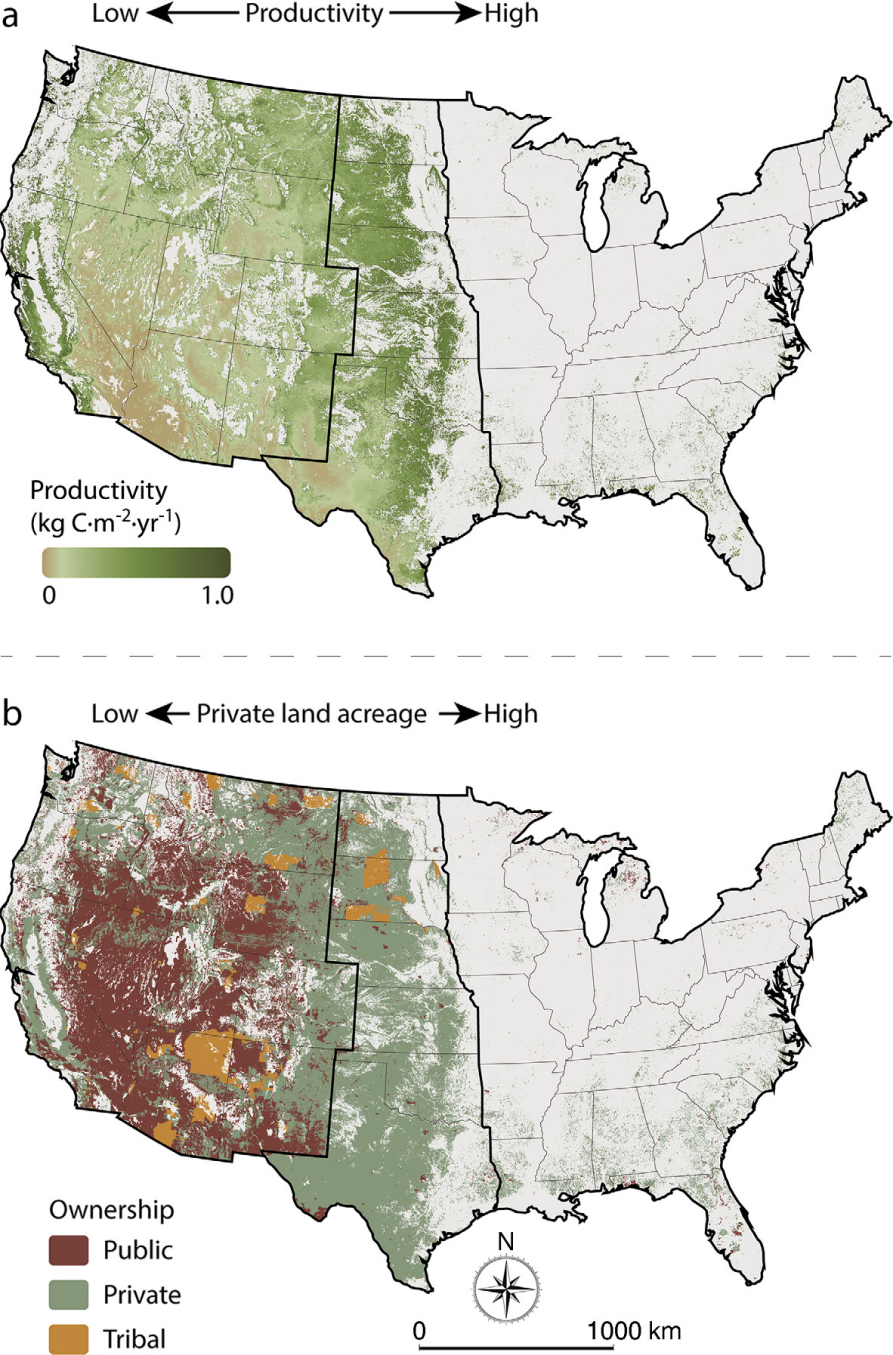
The distribution for rangelands across the coterminous United States (CONUS) showing the spatial patterns of (a) annual productivity and (b) land ownership. The heavy lines indicate the delineation of western states, Great Plains states, and eastern states.

**Figure 3:**
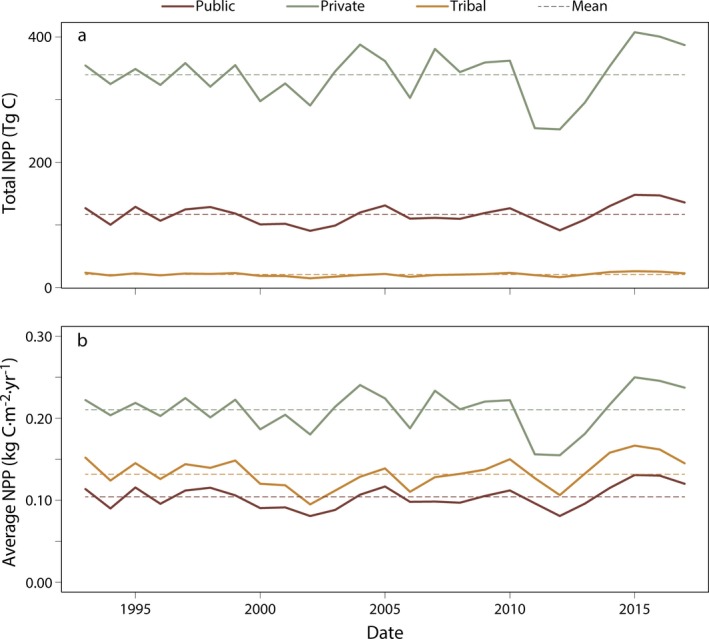
(a) Total production and (b) average productivity for rangelands across CONUS, 1993–2017. Total production and average productivity are consistently higher on private lands than public and tribal lands. Dashed lines represent mean values through time. Despite noticeable interannual variability, there are no significant temporal trends at the CONUS scale.

**Figure 4:**
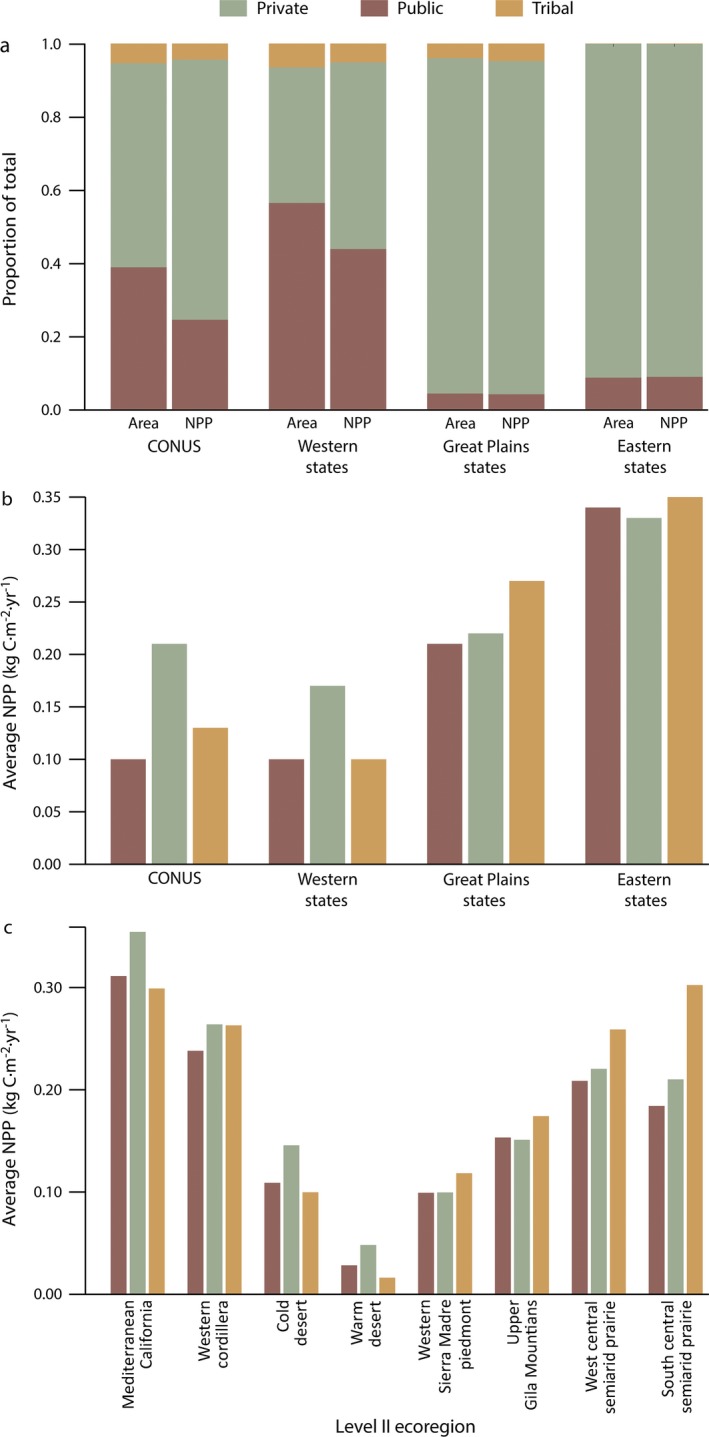
(a) Area and mean annual NPP (1993–2017) for each ownership class shown as proportions of total area and NPP across CONUS, western states, Great Plains states, and eastern states. Western states show a low proportion of NPP on public rangelands despite the high proportion of public rangeland area. (b) Average NPP by ownership across CONUS, western states, Great Plains states, and eastern states. (c) Average NPP by ownership across select EPA Level II ecoregions.

**Figure 5:**
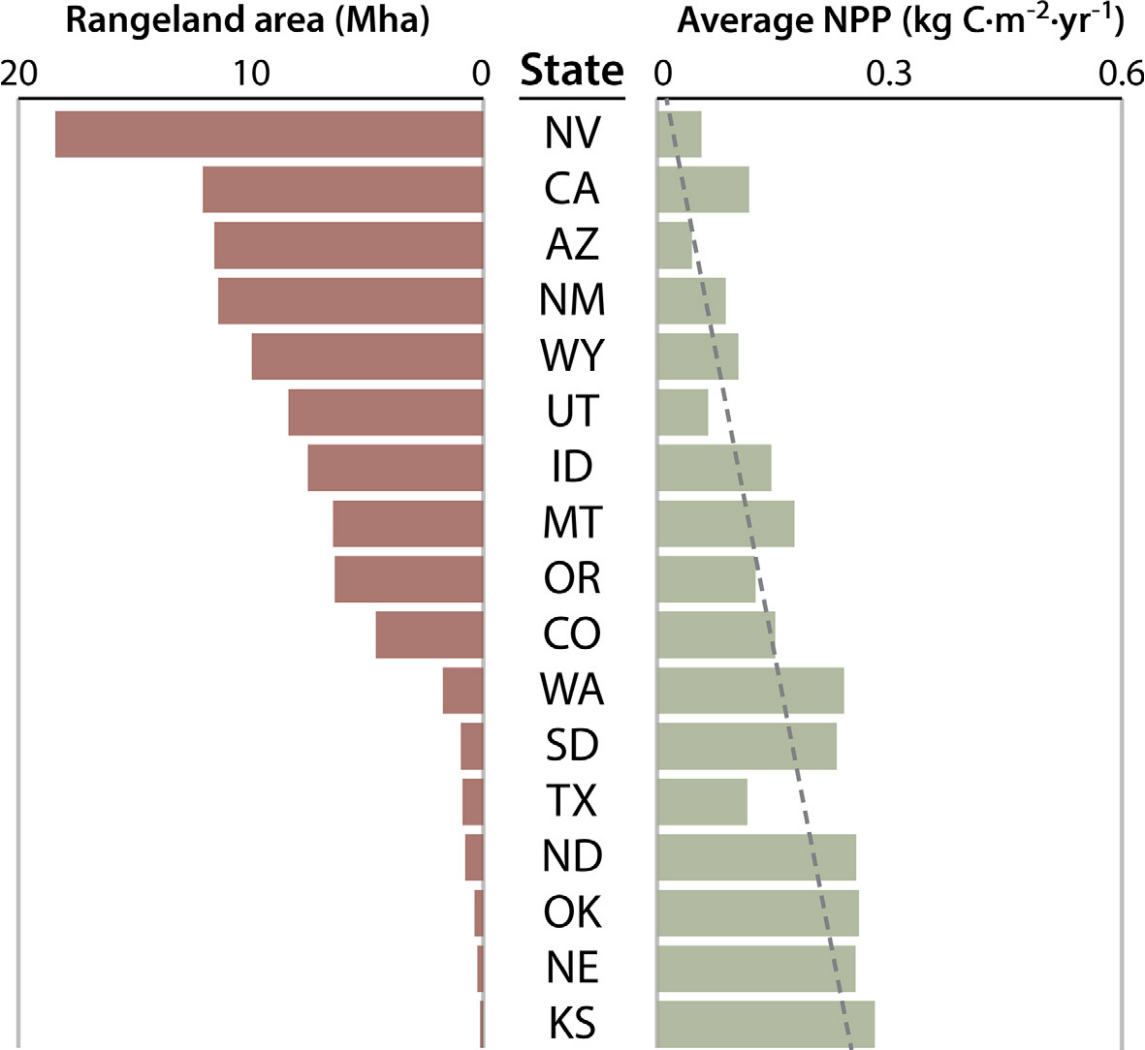
Ranking of public rangeland acreage and average productivity across western and Great Plains states. These states contain ~99% of the public rangeland across CONUS. Acreage of public rangeland is inversely related to average productivity (⍴ = −0.86; *P* < 0.01). States are NV, Nevada; CA, California; AZ, Arizona; NM, New Mexico; WY, Wyoming; UT, Utah; ID, Idaho; MT, Montana; OR, Oregon; CO, Colorado; WA, Washington; SD, South Dakota; TX, Texas; ND, North Dakota; OK, Oklahoma; NE, Nebraska; KS, Kansas.

Within CONUS, ~35% of the total land area is rangeland, with 95% of these rangelands occurring in the Great Plains and western United States (Fig. 2). NPP on rangelands is largely driven by climate, generally decreasing from east to west (Fig. 2a). In the Great Plains, nearly all (~91%) of the rangelands are privately owned, while in the western states most (~56%) are in the public domain. A small fraction (~5%) of CONUS rangelands are under Native American tribal jurisdiction (Fig. 2b). Across CONUS, both total and average vegetation production on private rangelands is double that of public and tribal lands (Appendix S1: Table S1; Fig. 3). While expected given the prevalence of private lands across eastern and Great Plains rangelands, subcontinental and ecoregional analyses also show that private and tribal rangelands are more productive than their public counterparts (Fig. 4). There were no significant trends in rangeland productivity at the CONUS scale regardless of ownership (1993–2017; Fig. 3). Interannual variation is present and likely reflects climate dynamics, particularly changes in annual precipitation. Droughts of the early 2010s are captured with sizable drops in total production and average productivity. These droughts occurred primarily within the Great Plains, where ownership is mostly private. Although productivity declined, these rangelands were drought resilient, returning to normal levels of productivity at both national and ecoregion scales (Fig. 3; Appendix S1: Fig. S1). At the state level, total area of public rangelands in each state is inversely related to average productivity of those rangelands (⍴ = −0.82; *P* < 0.01). Across the western and Great Plains states, where 95% of public rangelands occur, this dynamic is stronger (⍴ = −0.86; *P* < 0.01; Fig. 5).

Understanding the spatiotemporal dynamics and heterogeneity of NPP across private, public, and tribal rangelands enables greater understanding and execution of cross‐boundary conservation and management strategies. As socioecological systems, these dynamics of productivity are key to maintaining both the societal and ecological integrity of rangeland systems. Knowing that the vast majority of productivity is under private ownership and management will assist in structuring national conservation practices and programs to implement the right management, in the right place, with the right resources. Quality, not just quantity or size, is equally important (Pimm et al. 2018). Public rangelands do not capture the productivity or ecosystem services that depend on productivity to the degree that private rangelands do. Going hand in hand with private rangelands, tribal rangelands contribute to the overall heterogeneity of rangelands across the ecological mosaic, particularly in the western United States where ownership is more fragmented.

It is clear that maintaining rangeland productivity, which is vital for conserving broader ecological processes, ecosystem functions, and livelihoods, is about more than simply conserving acreage. Although conservation of large tracts of rangeland is beneficial, acreage alone does not capture or directly quantify the ecological functions, processes, or ecosystem services conserved. Incorporating strategies that include comprehensive ecosystem services and all components of ownership (public, private, and tribal) into broader conservation paradigms is critical for maintaining ecosystem function and the goods and services they provide. In regions or states where rangeland is mainly public and interspersed with private and tribal holdings (e.g., Nevada, California, Arizona, New Mexico, and Utah; Fig, 5) crafting novel approaches that include private, public, and tribal lands is paramount to conserving the acreage of public rangelands and the high productivity of private and tribal rangelands. In these regions, a sole focus of conserving public land through grazing restrictions can have unintended negative consequences on adjacent private land (Runge et al. 2018). In other areas where rangeland is predominantly private (e.g., Texas, Oklahoma, Nebraska, and Kansas; Fig. 5) a focus on private rangeland conservation is adequate. Additionally, while we only examined production dynamics in the U.S., ownership–production dynamics in other countries and geographies are equally relevant to rangeland conservation (Pimm et al. 2018). Those dynamics, however, may be strikingly different, not only due to ecological productivity, but also due to unique ownership patterns, policies, and histories. For example, across Africa the ownership–production dynamics have been shaped by complex histories of human migration, colonialism, and conservation and development policy heavily influenced by international institutions (Homewood 2004). In addition to different dynamics, the mechanisms for conservation will also differ, ranging from community‐based conservation initiatives to broad scale national and international policies and assistance.

Developing conservation strategies on U.S. private and tribal rangelands presents unique challenges as private landowners, whether individual or corporate, hold substantial liberties to manage land as they see fit. Management can be driven by a suite of factors that are more often than not socioeconomic rather than ecological. This is of particular importance across the western United States, where there is an accelerating transition away from ranching, toward more profitable forms of cultivation, subdivision, or development (Maestas et al. 2001, Hansen et al. 2002, Brunson and Huntsinger 2008). As little can be done through policy or regulation, voluntary, incentive‐based technical and financial support for private conservation is crucial in preserving America's rangelands. Furthermore, private rangeland provides unique opportunities for conservation effectiveness, as decision‐making can occur more efficiently and innovative strategies can be implemented more rapidly. Rangeland conservation programs that work collaboratively with private land owners, including federal and state agencies (e.g., NRCS), land trusts, non‐governmental organizations, and industry, can increase the economic viability of rangelands while maintaining ecological and societal values of the entire system (Havstad et al. 2007, Ferranto et al. 2012). To ensure that conservation objectives are successfully met and to demonstrate societal benefits, science must continually evaluate outcomes and provide guidance for future program implementation, irrespective of private, public, or tribal ownership.

## Data Availability

Data are available on the Dryad Digital Repository: https://doi.org/10.5061/dryad.pm929m9
