# Supplementary material for: Patterns of rangeland productivity and land ownership: Implications for conservation and management
Source: Ecol Appl. 2019 Feb 27;29(3):e01862. doi: 10.1002/eap.1862 (PMC6850440; doi:10.1002/eap.1862)
Supplement: Supplementary file 1 [file EAP-29-na-s001.pdf]

Supporting Information. Robinson, N. P., B. W. Allred, D. E. Naugle, and M. O. Jones. 2019. Patterns of rangeland productivity and land ownership: Implications for conservation and management. *Ecological Applications.*

Appendix S1

Table S1. Rangeland area and production dynamics, both total production and average productivity across ownership classes. Data are given for CONUS, the western states, the Great Plains states, and for eastern states and averaged from 1993–2017.

	**Area (Mha)**	**Area (%)**	**Production (Tg)**	**Production (%)**	**Productivity (kg c m^{-2} y^{-1})**
**CONUS**					
Public	112.51	0.39	117.06	0.24	0.1
Private	161.54	0.56	339.85	0.71	0.21
Tribal	15.72	0.05	21.03	0.04	0.13
	**289.77**	**1**	**477.94**	**1**	
**Western States***					
Public	107.43	0.56	104.32	0.44	0.1
Private	70.58	0.37	121.53	0.51	0.17
Tribal	12.43	0.07	12.28	0.05	0.1
	**190.44**	**1**	**238.13**	**1**	
**Great Plains States†**					
Public	3.51	0.04	7.43	0.04	0.21
Private	74.48	0.92	164.05	0.91	0.22
Tribal	3.26	0.04	8.68	0.05	0.27
	**81.25**	**1**	**180.16**	**1**	
**Eastern States‡**					
Public	1.57	0.09	5.31	0.09	0.34
Private	16.49	0.91	54.27	0.91	0.33
Tribal	0.02	<0.01	0.08	<0.01	0.35
	**18.08**	**1**	**59.66**	**1**	

[*] Washington, Oregon, California, Idaho, Nevada, Arizona, New Mexico, Utah, Colorado, Wyoming, & Montana

[†] N. Dakota, S. Dakota, Nebraska, Kansas, Oklahoma, & Texas

[‡] All other CONUS states

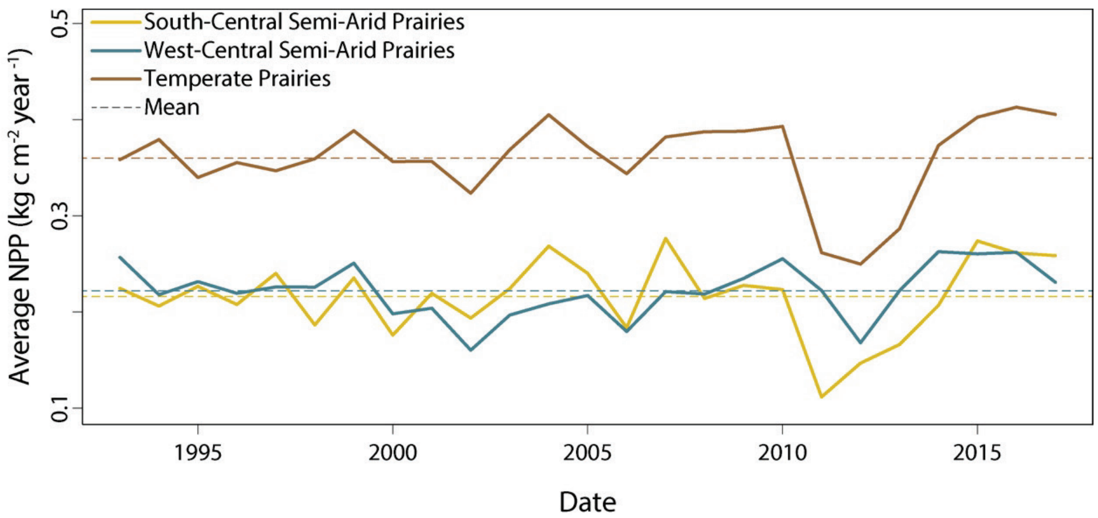

Fig. S1. Average productivity of private rangelands from 1993 to 2017 for select Level II Ecoregions within the Great Plains. These Ecoregions are dominated by private rangelands and were severely impacted by droughts of the early 2010s. The post drought recovery demonstrates the underlying resilience of these rangeland systems.
